# Immunoglobulin G Is Increased in the Injured Spinal Cord in a Sex- and Age-Dependent Manner

**DOI:** 10.1089/neu.2022.0011

**Published:** 2022-07-27

**Authors:** Andrew N. Stewart, Ethan P. Glaser, William M. Bailey, John C. Gensel

**Affiliations:** ^1^ Department of Physiology, University of Kentucky, Lexington, Kentucky, USA.; ^2^ Spinal Cord and Brain Injury Research Center, and University of Kentucky, Lexington, Kentucky, USA.; ^3^ College of Medicine, University of Kentucky, Lexington, Kentucky, USA.

**Keywords:** acute pathology, aging, secondary injury, sex differences

## Abstract

There are limited studies examining age and sex as biological variables in the pathophysiology of spinal cord injury (SCI). The use of older animals and sex-balanced groups in SCI models is increasingly prioritized to better match clinical demographics. Including older animals in SCI studies is technically challenging, and outcomes are unpredictable with respect to biological and treatment responses. Incidental discoveries that are unrelated to the question under investigation often emerge while including age and sex as biological variables. When probing tissue homogenates on Western blots of 4- and 14-month-old (MO) mice, we identified a sex- and age-dependent increase in immunoglobulin G (IgG) within the spinal cords of older, 14-MO mice acutely after SCI, with females having more IgG compared with males. We further probed to determine whether differences in hemorrhage exist between sexes or ages by evaluating hemoglobin within spinal homogenates. Differences in hemoglobin between sexes and ages were not consistently observed. Because IgG was elevated in an age- and sex-dependent manner without of evidence of differences in hemorrhage, our findings point to potential pre-existing differences in IgG within mouse plasma in an age- and sex-dependent manner. This report has identified age- and sex-dependent differences in infiltrating IgG into the injured spinal cord environment that may affect injury and recovery processes. Our findings highlight that systemic contributions to SCI can be sex- and age-dependent and illustrate the value of reporting incidental discoveries.

## Introduction

Older age at time of spinal cord injury (SCI) is associated with worse functional outcomes in both clinical populations and animal models.^1–8^ The narrative surrounding a decline in recovery with advanced age has been associated with decreased plasticity and neural adaptations to injury.^7,8^ There is an emerging understanding that underlying secondary injury cascades are also exacerbated with aging and contribute to worse functional outcomes.^4,5^ Advanced age at time of SCI causes larger lesions, increased accumulation of oxidative damage, and more reactive oxygen species production by macrophages.^2–5,9^ Identifying biomarkers of injury that are exacerbated with aging will help tailor treatment approaches appropriate for a diverse clinical demographic.

Physiological adaptations to aging, and as a function of sex, interact to change the pathophysiology of SCI.^9,10^ As more research is performed that considers age and sex as biological variables in neurotrauma, unexpected findings regarding biological contributions to injury are emerging.^10^ For example, our past work targeted an age-dependent increase in oxidative stress using 2,4-dinitrophenol as a mild mitochondrial uncoupler that resulted in reciprocal effects in younger and older mice.^2^ Specifically, younger mice experienced toxicity while older mice experienced benefit from this treatment strategy.

While debated, biological responses to SCI also differ in a sex-dependent manner, with several studies indicating that males have worse recovery after SCI.^10^ Inflammation after neurotrauma is emerging as a sex-dependent regulator of injury and recovery. Microglia proliferation is increased in male versus female mice acutely after both traumatic brain injury and SCI,^9,11^ while the macrophage response in females exhibits transcriptional profiles indicative of greater reactivity.^9^ Taken together, our past work has validated that both age and sex change the underlying biology of SCI, which can have important implications for treatment strategies and responses.

Through the inclusion of both sex and age as biological variables in SCI research, many incidental findings emerge outside of the primary question under investigation. For example, we have found that older male mice experience a disproportional increase in death after SCI as well as a sex- and age-dependent decrease in red blood cells within the plasma after injury.^10^ These findings are often difficult to publish on their own because of the incidental nature, but are nevertheless important and meaningful for our emerging understanding of personalized medicine for SCI.

Here we report an incidental discovery in the context of a hypothesis-driven study examining antioxidant defenses in young (4-month-old [MO]) and aged (14-MO) male and female mice after SCI.^30^ Specifically, the blood-borne protein, immunoglobulin G (IgG), is increased in a sex- and age-dependent manner within the spinal cord acutely after SCI. This discovery points to a new potential mechanism contributing to the age-dependent pathology in neurotrauma.

## Methods

### Animals and surgical procedures

Note: Data were obtained while conducting larger, hypothesis-driven experiments for the companion publication and data from this report were not presented in the companion article.^30^ All methods and procedures were approved by the University of Kentucky's Institutional Animal Care and Use Committee. Male and female C57Bl/6 mice of ages 4-MO (Jackson Laboratories), and 14-MO (National Institute on Aging) were utilized in these experiments. Mice received either a T9 laminectomy only or a T9 laminectomy with a 60 kDyn spinal contusion^12^ (Infinite Horizons Impactor), or naïve mice were used with no injury.

**FIG. 1.**
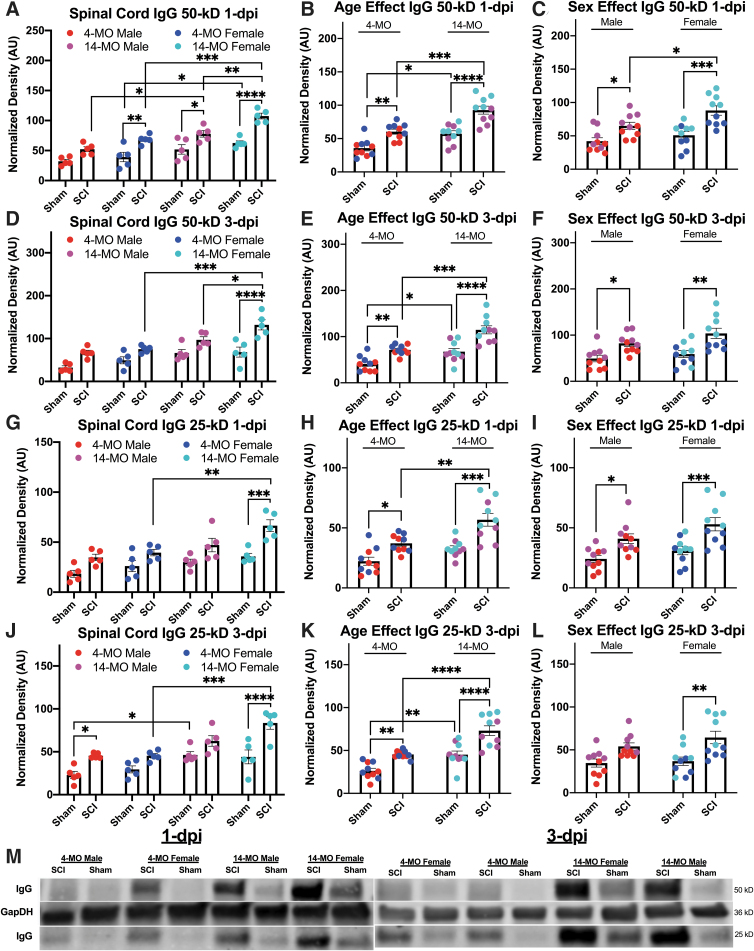
Age, sex, and spinal cord injury (SCI) increase immunoglobulin G (IgG) within the spinal cord at one and three days post-injury (dpi). The IgG 50- and 25-kD were detected on Western blots using secondary antibodies targeting mouse IgG. 14-month-old (MO) female mice had significantly more 50 kD IgG compared with sham-injured-, age-, and sex-matched comparisons at both one dpi (**A**), and three dpi (**D**). After collapsing groups across sexes, 14-MO mice had significantly more 50 kD IgG compared with 4-MO mice at both one dpi (**B**) and three dpi (**E**). After collapsing across ages, both males and females had a significant increase in 50 kD IgG at both one dpi (**C**) and three dpi (**F**) with female mice having more 50 kD IgG compared with male mice after SCI. Assessment of the 25 kD IgG band revealed similar patterns as the 50 kD band, specifically finding that older female mice had a significant injury- and age-induced increase in IgG at both one dpi and three dpi (**G,J**). Similarly, there was a main effect of age that, on collapsing across sexes, resulted in significant increases in 25 kD IgG in 14- compared with 4-MO mice after SCI at both one dpi and three dpi (**H,K**). Similarly, a three-way analysis of variance (ANOVA) did reveal a main effect of sex for 25 kD IgG. In contrast to 50 kD IgG, there was no difference between female- and male-injured mice (**I,L**). Analyses were performed using three-way ANOVA (A,D,G,J) using Sidak pairwise comparisons to assess differences between all groups deviating by one variable in both the three-way ANOVA design as well as after collapsing across sex or age. Independent variables of sex and age were collapsed and assessed to determine whether the significant main effects were found between groups receiving SCI. All error bars represent standard error of the mean. Data represent square root-transformed values normalized to GAPDH. Blots depict (**M**) representative images obtained at 50 kD (IgG heavy chain), 25 kD (IgG light chain), and 36 kD (GAPDH). *n* = 5 (A,D,G,J) or *n* = 10 (B,C,E,F,H,I,K,L). *p < 0.05, **p < 0.01, ***p < 0.001, ****p < 0.0001.

**FIG. 2.**
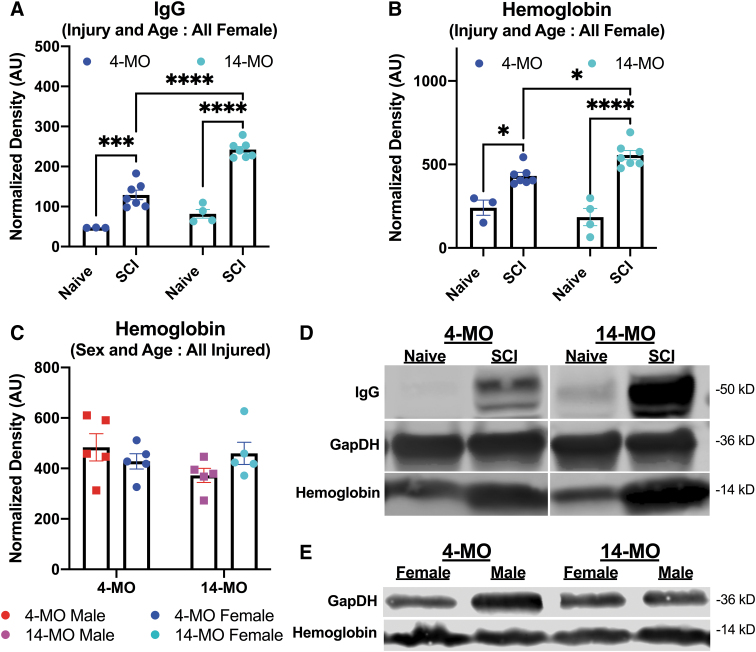
Immunoglobulin G (IgG) but not hemoglobin is consistently increased with older age in injured spinal cords at one day post-injury (dpi). The IgG and hemoglobin were detected in a separate cohort of all female mice on Western blots at one dpi (**A,B,D**). 14-month-old (MO) female mice had significantly more IgG (A) and hemoglobin (B) compared with sham-injured- and age-matched comparisons. Effects observed in IgG replicated results found in Fig. 1; however, a second replicate of hemoglobin to detect sex and age differences did not replicate an increase in hemoglobin in 14-MO mice with older age (**C**). Analyses were performed using two-way analysis of variance, using Sidas pairwise comparisons to assess differences between all groups deviating by one variable. All error bars represent standard error of the mean. Blots depict **(D,E)** representative images obtained at 50 kD (IgG heavy chain), 36 kD (GAPDH), and 14 kD (hemoglobin) at one dpi. Data represent square root-transformed values normalized to GAPDH. (A,B) *n* = 3-4 (naïve) or *n* = 7 (SCI), or (C) *n* = 5/group. *p < 0.05, ***p < 0.001, ****p < 0.0001.

Surgeries were performed under ketamine (100.0 mg/kg) and xylazine (10 mg/kg) anesthesia, and mice were given buprenorphine slow-release formulation (Buprenex SR; 1.0 mg/kg), saline (1.0 mL/day), and enrofloxacin (antibiotic; 5.0 mg/kg) after SCI. Saline (1.0 mL) and enrofloxacin (5.0 mg/kg) were provided daily after injury for five consecutive days. Bladders were manually expressed 2 × /day for the duration of the study. Data obtained for Figures 1 and 2 of this article were collected from the same animals used for the data in Figures 2–4 and 5 (respectively) of our companion article. Animals from Fig. 2A,B of this article were either given no treatment, or 250 μL saline (ip) as a vehicle for the experimental drug from our companion article.^30^

### Protein preparation

Protein was isolated from whole spinal homogenates from mice at 24 h or 72 h post-injury. To isolate spinal cords, mice were anesthetized using an overdose of ketamine (4.0–5.0 mg) and xylazine (0.4–0.5 mg), followed by transcardial perfusion using ice cold 0.1 M phosphate buffer (PB) to remove blood within the circulation. Then 6.0 mm of tissue, centered on the lesion epicenter, was isolated and immediately homogenized using a Dounce homogenizer in PB containing protease inhibitors (Complete Mini, EDTA-FREE; 11836170001; Sigma Aldrich). Homogenates were then sonicated on ice before addition of radioimmunoprecipitation assay (RIPA) buffer, which does not contain sodium dodecyl sulfate (SDS; 20-188; Sigma Aldrich). Samples were incubated in RIPA buffer for 20 min before pelleting debris.

Protein concentration was determined using bicystronic acid assays (BCA; 23225; ThermoFisher). All samples were diluted to 2.0 mg/mL in Laemmli Buffer (1610747; Bio-Rad) prepared with 2-marcaptoethanol at a final concentration of 5%. Samples were boiled for 5 min before allocation and storage at -80°C.

### Western blotting

Western blots were performed under reducing conditions using pre-made 4–20% gradient gels (4561096; Bio-Rad). A total of 30 mg of protein was loaded into each well, and samples were run in Tris/Glycine/SDS buffer (1610732; Bio-Rad) at 90 mV for 30 min, then 120 mV until the indicator dye reached the bottom of the gel. Membranes were acclimated to the Tris/Glycine buffer (161073; Bio-Rad) containing 20% methanol for 15 min before transferring onto nitrocellulose membranes (1620233; Bio-Rad) at 300 mA for 90 min.

For the first experiment (Fig. 1), when more samples were run than available wells, multiple blots were performed with each blot having all groups represented on the blot, with one well containing a sample of liver protein on each blot to normalize transfer efficiencies. For the second experiment (Fig. 2), all samples from within an age were run on separate blots because the intent was to identify injury effects. For both experiments, all blots within an experiment were run, transferred, stained, and imaged at the same time in the same conditions. After transfer, all blots were immersed in 5% milk dissolved in Tris-buffered saline (TBS) for 30 min to block unspecific binding.

Primary antibodies were diluted in TBS containing 0.1% Tween-20 (TBST) and incubated at room temperature overnight, and secondary antibodies were also diluted in TBST and incubated for 1 h at room temperature. Blots were washed 3** ×** using TBST after primary and secondary antibody incubations. Before imaging, blots were washed in TBS to remove residual Tween-20 that causes background on fluorescence imaging. Imaging of blots was performed using fluorescent development (Odyssey CLx; LI-COR Biosciences), and all blots within an experiment were imaged in the same exposure event.

Because these findings emerged incidentally with other ongoing work,^30^ IgG bands were detected after probing for other primary antibodies. Specifically, for the first experiment (Fig. 1), we evaluated for gamma-glutamyl cysteine ligase (GCLC; mouse anti-GCLC; 1:1,000; H00002729-M01; Novus Biologicals), which is observable at 70 kD (not reported). Secondary antibodies against mouse IgG (1:10,000; goat anti-mouse IgG IRDye-680; LI-COR Biosciences) subsequently revealed the IgG bands of interest at 50 and 25 kD. Both the 50- and 25-kD bands were used for analyses.

For the second experiment (Fig. 2), hemoglobin was revealed using primary antibodies against hemoglobin (rabbit anti-hemoglobin; 1:1,000; PA5-97559; ThermoFisher Scientific) and detected at 14–16 kD using fluorescent secondary antibodies (1:10,000; goat anti-rabbit IgG IRDye-800; LI-COR Biosciences). Because differences were found in hemoglobin in the second experiment, injured samples from the first experiment were run again to validate findings in a second cohort of mice.

After imaging, blots were probed using a primary antibody against GAPDH as a housekeeping gene. Blots were incubated again overnight in primary antibody (rabbit anti-GAPDH; 1:5,000; ab9484; Abcam Co.) followed by secondary antibody incubation and fluorescent development as just described. For our second experiment that sought to identify hemoglobin within the injured spinal cords, IgG was revealed intentionally using only secondary antibodies targeting mouse IgG to reproduce findings on a second cohort of mice.

### Statistics

Optical densities of blots were quantified using LI-COR Biosciences Image Studio Lite. First, the consistent liver sample on each blot was used to normalize values between blot densities, followed by normalizing signal intensities to the GAPDH housekeeping gene. The GAPDH intensities were normalized to a consistent value, followed by normalization of the gene of interest to the same proportion. For IgG values obtained at one and three days post-injury (dpi), male and female, 4- and 14-MO, sham and injured groups (*n* = 5/group) were evaluated using three-way analysis of variance (ANOVA). Because we were underpowered to detect all pairwise comparisons, effects of age or sex were also evaluated in collapsed groups.

Data from Fig. 2 were detected as part of an ongoing experiment (*n* = 3-4/group for naïve controls, *n* = 7/group for SCI-mice) that used all female mice of ages 4- and 14-MO at one dpi, so sex was not included as a biological variable. Evaluation of hemoglobin on the first cohort of mice was later performed and used only mice receiving SCI of both 4-MO and 14-MO, male and female mice. Detection of hemoglobin was evaluated using two-way ANOVA for both experiments. All pairwise comparisons were performed using the Sidak method of *post hoc* testing. For all analyses, data were square root transformed to conform to heteroscedasticity of variance and normal distribution. Data are presented as square root transformed values. Alpha levels of *p =* 0.05 or less were considered significant.

## Results

### IgG is increased in a sex-, age-, and injury-dependent manner after SCI

Hemorrhage after spinal contusion exacerbates secondary injury. Blood and plasma proteins enter the central nervous system (CNS) parenchyma and reside until being broken down and cleared. During tissue processing, vascular blood is cleared after transcardial perfusion leaving only the plasma proteins residing within the CNS parenchyma. First, to ensure that there were no pre-existing differences in impact parameters between groups that could explain differences in injury severity, we assessed the force and displacement values obtained from the impactor during injury. Two-way ANOVA did not reveal any main effect of sex or age for injury force (sex, F_(1,35)_ = 1.56, *p* = 0.22; age, F_(1,35)_ = 0.37, *p* = 0.54) or displacement (sex, F_(1,35)_ = 0.38, *p* = 0.53; age, F_(1,35)_ = 0.17, *p* = 0.67), confirming that injury parameters did not differ by sex or age. Mean values, standard deviations, and sample sizes obtained are presented in Table 1.

**Table 1.** Force and Displacement Values

	4-MO			14-MO		
	Mean	SD	n	Mean	SD	n
Force						
Female	64.44	2.70	^**^9	68.90	^*^15.02	10
Male	64.10	3.90	10	62.80	1.99	10
Displacement						
Female	577.67	167.00	^**^9	578.20	156.78	10
Male	534.20	87.69	10	569.20	101.40	10

Data represent averages from both one day post-injury (dpi) and three dpi experiments. ^*^One mouse had a dura rupture on impact causing a spike in the force estimate to 111 kDyn. This did not affect the molecular assessments in this mouse. Data for 50- and 25-kD IgG fell in the middle of the range for this group. ^**^Force and displacement values were lost for one mouse. SD, standard deviation.

Next, we found that the 50-kD band of IgG is significantly elevated within the spinal cords after injury at both one and three dpi (F(_1,32_) = 63.60, *p* < 0.0001; F(_1,32_) = 46.41, *p* < 0.0001, respectively [Fig. 1]). Further, both increased age and being female result in significantly more detectable 50-kD IgG at one and three dpi (one dpi: age, F(_1,32_) = 51.04, *p* < 0.0001 ; sex, F(_1,32_) = 17.85, *p* < 0.001) (three dpi: age, F(_1,32_) = 37.56, *p* < 0.0001; sex, F(_1,32_) = 7.36, *p* < 0.01).

To probe the main effects of age and sex found in the three-way ANOVA models, sex and age were collapsed, and two-way ANOVAs were used to assess where main effects of sex and age were found. After combining sexes, 50-kD IgG was increased after SCI in both 4- (one dpi, *p* < 0.01; three dpi, *p* < 0.01) and 14-MO mice at both time points (one dpi, *p* < 0.0001; three dpi, *p* < 0.0001). After combining ages, 50-kD IgG was increased after SCI in both female (one dpi, *p* < 0.001; three dpi, *p* < 0.001), and male mice at both time points (one dpi, *p* = 0.05; three dpi, *p* < 0.05). Importantly, 14-MO mice had more 50-kD IgG after SCI compared with 4-MO mice at both one dpi (*p* < 0.001) and three dpi (*p* < 0.001), and female mice had more 50-kD IgG after SCI compared with male mice (*p* = 0.05).

At the level of pairwise comparisons with non-collapsed groups, 14-MO female mice had a significant increase in 50-kD IgG after SCI compared with age-matched males at both one dpi (*p* < 0.01) and three dpi (*p* < 0.05). The 50-kD IgG was increased in both 14-MO males (*p* < 0.05) and females (*p* < 0.001) compared with sex-matched 4-MO mice at one dpi, and only 14-MO females had increased 50-kD IgG relative to sex-matched 4-MO mice at three dpi (*p* < 0.001). Significant injury effects were found for both 14-MO males (*p* < 0.05) and females (*p* < 0.0001) as well as 4-MO females (*p* < 0.01) at one dpi, but a significant injury effect was only found for 14-MO females at three dpi (*p* < 0.0001) (Fig. 1). Significant, experimental effects of an age- (F(_1,17_) = 43.79, *p* < 0.0001 ) and injury-dependent (F(_1,17_) = 117.40, *p* < 0.0001) increase in 50-kD IgG were replicated in a second cohort of mice at one dpi in a subsequent experiment (Fig. 2A).

When evaluating the 25-kD IgG most effects were comparable, but a few significant comparisons were lost likely because of the 25-kD band showing less of a signal. The 25-kD band of IgG is also significantly elevated within the spinal cords after injury at both one and three dpi (F(_1,32_) = 36.68, *p* < 0.0001; F(_1,32_) = 41.33, *p* < 0.0001, respectively [Fig. 1]). Similar to the 50-kD band, both increased age and being female result in significantly more detectable 25-kD IgG at one dpi, but only an effect of age was found at three dpi (one dpi: age, F(_1,32_) = 22.50, *p* < 0.0001; sex, F(_1,32_) = 8.72, *p* < 0.01) (three dpi: age, F(_1,32_) = 40.52, *p* < 0.0001; sex, F(_1,32_) = 2.943, *p* = 0.10).

After combining sexes, 25-kD IgG was increased after SCI in both 4-MO (one dpi, *p* < 0.05; three dpi, *p* < 0.01) and 14-MO mice at both time points (one dpi, *p* < 0.001; three dpi, *p* < 0.0001). After combining ages, 25-kD IgG was increased after SCI in both female (one dpi, *p* < 0.01; three dpi, *p* < 0.001), and male mice at one dpi but not male mice at three dpi (one dpi, *p* < 0.05; three dpi, *p* = 0.09). Importantly, 14-MO mice had more 25-kD IgG after SCI compared with 4-MO mice at both one dpi (*p* < 0.01) and three dpi (*p* < 0.0001), but there were no differences between male or female mice with SCI at either one dpi or three dpi (one dpi, *p* < 0.24; three dpi, *p* < 0.70).

Pairwise comparisons in non-collapsed groups, revealed 14-MO female mice had a non-significant increase in 25-kD IgG after SCI compared with age-matched males at both one dpi (*p* = 0.056) and three dpi (*p* = 0.085). The 25-kD IgG was increased in 14-MO females (one dpi, *p* < 0.01; three dpi, *p* < 0.001) and but not males (one dpi, *p* < 0.53; three dpi *p* = 0.26) compared with sex-matched 4-MO mice at both one dpi and three dpi. Significant injury effects were found for only 14-MO females at one dpi (*p* < 0.001) and three dpi (*p* < 0.0001 (Fig. 1), a well as 4-MO males at three dpi (*p* = 0.05). Significant, experimental effects of an age- (F(_1,17_) = 26.47, *p* < 0.0001) and injury-dependent (F(_1,17_) = 70.06, *p* < 0.0001) increase in 25-kD IgG were replicated in a second cohort of mice at one dpi in a subsequent experiment (Fig. 2).

Collectively, the major novel conclusion from these findings indicates that being older and female increases IgG within the spinal cords after SCI and these effects can be detected at both one dpi and three dpi across different molecular weights of IgG (Fig. 1).

### Effects of increased age on hemoglobin are variable between experimental replicates post-SCI

We observed that IgG increased in an age-dependent manner across three cohorts of mice and two time points. Next, we probed protein obtained from both experiments for hemoglobin to determine whether more blood had entered the spinal cords in older or female mice post-SCI. Because our observation of increased IgG was discovered incidentally in the context of a larger study, tissue for hemoglobin analyses was limited, and hemoglobin was first examined in the second experiment utilizing all female mice.

In experiment 1, we observed that hemoglobin is also increased in the spinal cord with a significant injury-by-age interaction (F(_1,17_) = 6.84, *p* < 0.05). While significant main effects of injury were observed (F(_1,17_) = 65.98, *p* < 0.0001), there was no main effect of age (F(_1,17_) = 0.98, *p* < 0.33). Sidak pairwise comparisons revealed that hemoglobin was significantly increased in both 14- (*p* < 0.0001) and 4-MO mice at one dpi (*p* < 0.01), with 14-MO injured mice having significantly more hemoglobin than 4-MO injured mice (*p* < 0.05; Fig. 2B).

Next, we ran more protein from the first experiment (from Fig. 1) to replicate findings in another cohort of mice, as well as test for sex differences. We limited our samples to only mice receiving SCI to have equal group representation on each blot/experimental batch. In contrast to Fig. 2B, an increase in hemoglobin after SCI did not replicate as a main effect of age, nor were main effects of sex found at one dpi or sex (F(_1,16_) = 0.16, *p* = 0.69) at one dpi (Fig. 2C). Further, no differences were found in any pairwise comparison.

## Discussion

This report has identified increased IgG within the spinal cords of older and female mice after SCI even in samples with no evidence of differences in hemorrhage between groups. The IgG and other bloodborne proteins enter into the spinal cord from the plasma after injury. Infiltrating neutrophils after injury continue to break down the blood–brain barrier through secretion of extracellular matrix remodeling proteins such as matrix metallo-proteases.^13^ A breakdown of the blood–brain barrier sustains permeability between the injured spinal cord and plasma and allows for the exchange of proteins that are normally foreign to the CNS environment, such as IgG. While identifying IgG and hemoglobin within the spinal cord after injury is already known, observing a sex- and age-dependent increase in IgG is unexpected.

The role of IgG in the injured CNS environment is not well described but is not physiologically inert.^14–17^ The presence of acute IgG influx after neural injury may aid in clearing myelin and cellular debris^18^ to aid in recovery processes. Previous work, however, has also implicated IgG after SCI as a pathological driver of injury^14^ and has found auto-antibodies against CNS proteins in as little as five days post-SCI in humans.^19^ Ankeny and colleagues (2009)^14^ directly injected IgG obtained from mice with SCI into the spinal cords of naïve mice and found that a single injection is sufficient to induce complement activation and secondary injury large enough to cause paralysis in otherwise uninjured mice. After IgG binds to its target antigen and forms immune complexes, the IgG protein undergoes a conformational shift revealing a C1q binding site that activates the complement cascades.^20,21^ In the studies by Ankeny and colleagues,^14^ IgG obtained from naïve mice did not induce dysfunction when injected into healthy cords, and injection of IgG from naïve mice into injured spinal cords was not performed to evaluate for the role of circulating IgG on SCI pathology at the time of injury.

The IgG was obtained from mice at 42 dpi, which demonstrates that autoantibodies produced against CNS proteins form after SCI and contribute to ongoing pathology. The ability for infiltrating IgG at acute time points to bind to myelin debris and activate complement cascades is less well understood. Endogenous IgG that accumulates acutely after neural injury may have an ability to bind to damaged cell debris before the development of an adaptive auto-antibody response that develops against CNS proteins within weeks after injury.^18^

While we do not know exactly why IgG is increased in older and female mice after SCI, we posit two possible explanations. First, both older and female mice have been found to have increased IgG within their plasma in naïve conditions,^22,23^ therefore resulting in more total IgG infiltration after SCI. Next, while it is possible for an increase in plasma IgG to account for our findings within the spinal cord, older mice could also have experienced more hemorrhage. In one of our experimental replications, we observed an increase in IgG within plasma obtained from older mouse homogenates, which would argue for increased hemorrhage at older age. This was not replicated in a second cohort of mice, however. Despite not observing more hemoglobin within homogenates in one of our replications, an age- and sex-dependent increase in IgG persisted in the same samples. It is therefore more probable that an age- and sex-dependent increase in IgG is closer related to pre-existing differences within the plasma before injury.

Alternatively, sex- and age-dependent changes in blood–brain barrier permeability could account for increased plasma proteins after SCI. Age affects astrocyte responses after traumatic brain injury, specifically in the acute phase of injury, resulting in less astrocyte reactivity and end-feet around vasculature.^24^ If similar effects occur after SCI, a breakdown of the blood–brain barrier, independent of hemorrhage, could account for the observations in the current article, especially considering the observation of sex- and age-dependent increase in resting plasma IgG.^22^ Future work should evaluate whether this age-dependent increase in IgG affects debris clearance or inflammatory responses locally within the spinal cord; both are critical in the pathophysiology of SCI.

We evaluated SCI impact parameters that could explain differences in the initial force or tissue mechanics of injury but did not detect any differences between groups. While there were no differences in injury parameters in these experiments, we previously found that older mice have larger lesions and slightly less tissue sparing surrounding the lesion corresponding to worse functional outcomes.^2–5^ We also identified that macrophages infiltrating into SCI lesions of older animals have a more aggressive transcriptional profile and produce more reactive oxygen species, which results in more oxidative damage by seven dpi.^2,4,5,9^ Whether or not this age-dependent increase in IgG compounds secondary injury mechanisms, or interferes with recovery, has yet to be determined.

Our finding of increased IgG in older mice after SCI is congruent with recent reports by our laboratory, as well as others, which have identified age-dependent changes in the pathophysiology of SCI.^2–10,25,26^ Specifically, older age at time of injury results in larger lesions and worse functional recovery that is at least partially driven by increased oxidative stress derived from macrophages.^3–5^ While we do not yet know the nature of this increased IgG in females and with older age, the current findings provide yet another example of how the biology of SCI is both sex- and age-dependent.

There have been several recent reports that discuss how peripheral organ dysfunction contributes to SCI pathophysiology.^27,28^ Our findings raise an important concern that an aging physiology outside of the spinal cord can affect the injured microenvironment in potentially significant ways. Corroborating this notion, our previous reports have also identified that peripherally derived macrophages undergo age-dependent alterations that exacerbate damage once in the SCI environment.^5^ Further, several proteins within plasma that contribute to the pathophysiology of SCI also change their abundance with age, such as terminal complement proteins.^29^ Findings in our current report raise questions about how an aging organism as a whole can affect injury processes within the CNS environment.

Results presented here were not generated with the intent on identifying differences in IgG or hemoglobin within the spinal cord; our discoveries were incidental in nature. Our findings were observed during work from our companion article^30^ when performing Western blots to identify the redox response to SCI. It is essential to publish incidental findings such as those presented in this report to bring attention to meaningful discoveries that do not otherwise fit within the scope of a larger study. Incidental discoveries in this report will hopefully call attention to meaningful data that would otherwise be supplemental in our companion article.

## Conclusions

Our finding of increased IgG within the spinal cords of female and aged mice after SCI validate that sex and age affect the acute pathophysiology of injury. Future work should elucidate the functional repercussions of acutely elevated IgG to secondary injury and recovery, as well as identify other peripheral contributions to SCI that change with age.
